# Rationale, design and methods for a randomised and controlled trial to investigate whether home access to electronic games decreases children's physical activity

**DOI:** 10.1186/1471-2458-9-212

**Published:** 2009-06-29

**Authors:** Leon M Straker, Rebecca A Abbott, Jan P Piek, Clare M Pollock, Peter S Davies, Anne J Smith

**Affiliations:** 1 School of Physiotherapy, Curtin University of Technology, Perth, Australia; 2 School of Human Movement Studies, The University of Queensland, Brisbane, Australia; 3 School of Psychology, Curtin University of Technology, Perth, Australia; 4 Children's Nutrition Research Centre, The University of Queensland, Brisbane, Australia; 5 Curtin Health Innovation Research Institute, Curtin University of Technology, Perth, Australia

## Abstract

**Background:**

Many children are reported to have insufficient physical activity (PA) placing them at greater risk of poor health outcomes. Participating in sedentary activities such as playing electronic games is widely believed to contribute to less PA. However there is no experimental evidence that playing electronic games reduces PA. There is also no evidence regarding the effect of different types of electronic games (traditional sedentary electronic games versus new active input electronic games) on PA. Further, there is a poor understanding about how characteristics of children may moderate the impact of electronic game access on PA and about what leisure activities are displaced when children play electronic games. Given that many children play electronic games, a better understanding of the effect of electronic game use on PA is critical to inform child health policy and intervention.

**Methods:**

This randomised and controlled trial will examine whether PA is decreased by access to electronic games and whether any effect is dependent on the type of game input or the child's characteristics. Children aged 10–12 years (N = 72, 36 females) will be recruited and randomised to a balanced ordering of 'no electronic games', 'traditional' electronic games and 'active' electronic games. Each child will participate in each condition for 8 weeks, and be assessed prior to participation and at the end of each condition. The primary outcome is PA, assessed by Actical accelerometers worn for 7 days on the wrist and hip. Energy expenditure will be assessed by the doubly labelled water technique and motor coordination, adiposity, self-confidence, attitudes to technology and PA and leisure activities will also be assessed. A sample of 72 will provide a power of > 0.9 for detecting a 15 mins difference in PA (sd = 30 mins).

**Discussion:**

This is the first such trial and will provide critical information to understand whether access to electronic games affects children's PA. Given the vital importance of adequate PA to a healthy start to life and establishing patterns which may track into adulthood, this project can inform interventions which could have a profound impact on the long term health of children.

**Trial registration:**

This trial is registered in the Australia and New Zealand Clinical Trials Registry (ACTRN 12609000279224).

## Background

Nationally and internationally, promoting physical activity (PA) has become a major health priority. In the USA, Healthy 2010 has PA ranked as a leading health indicator [1] and the Department of Health in the UK have recently launched their "At least five a week" PA call for action [2]. In Australia, the Strategic Inter-Government forum on Physical Activity and Health was established in 1999 as the collaborative body to coordinate a national approach in supporting health-promoting PA in Australia. The resultant "Be Active Australia" framework was endorsed in 2005. Increasing PA is now a priority issue of state governments across Australia (eg  and ) with Queensland even calling 2008 "The year of Physical Activity" .

PA in adulthood is an important inverse risk factor for the major causes of mortality and morbidity including heart disease, stroke, cancer, musculoskeletal disorders, depression, obesity and diabetes [3]. Insufficient PA contributes a major international health burden [4-6] with Australian direct health care costs estimated at more than $400 million p.a. [7]. In Australia, insufficient PA has been estimated to account for 8,000 deaths per year and is the 4th leading cause of premature death, after obesity, tobacco and hypertension [8].

Whilst the data are inconclusive as to whether PA levels in children track through to adulthood [9], there is evidence to suggest that inactivity tracks [10]. Furthermore, lower levels of PA in childhood have been linked in the short-term with increased levels of obesity, poorer skeletal health [11], and poorer psychosocial well-being [12]. Whilst there is an absence of definitive data providing a causal link between PA and health, Biddle et al. conclude that the evidence suggests that PA in childhood has beneficial effects on cardiovascular disease, obesity, psychosocial outcomes, type II diabetes and osteoporosis [13].

In response to the growing awareness of the health benefits of PA, developed countries have established specific guidelines for PA by children [14-18], with Australia releasing their own in 2004 [19]. In a state-wide survey of 3,691 Queensland children we recently found that nine in ten children, on self-report, failed to meet PA guidelines of accumulating at least 60 minutes of moderate to vigorous activity every day [20]. Other surveys have reported smaller though still substantial proportions of Australian children are insufficiently active [21], with the state-wide WA study finding 1 in 4 high school boys and 1 in 3 high school girls reporting no PA [22]. Sufficient PA is clearly a critical aspect of getting a healthy start to life, yet many Australian children are not sufficiently physically active.

Increasing use of screen based media (SBM) is widely blamed for the perceived reduction of childhood levels of PA [23-25]. SBM exposure includes watching the television (TV), using computers and playing electronic games. TV viewing by children includes watching programs on free to air and pay TV and watching VHS/DVD videos. Computer use by school children includes searching for information on CD ROMs or the Internet, preparing documents and presentations, literacy/numeracy/problem solving activities, email and chat communication. Electronic games are played on computers, dedicated hand held devices (such as Nintendo DS and PSP) and consoles viewed on TV (such as PlayStation, Wii and Xbox).

Nearly all children in Australia now use SBM. Australian Bureau of Statistics' figures show 98% of school aged children watch TV, 95% use computers and 71% play electronic games (compared to 75% who read for leisure) [26]. Children's exposure to SBM starts at an early age. We recently reported that by 5 years of age over half of Western Australian children are using a computer [27]. SBM use is not only very prevalent, but daily doses are now substantial. Marshall et al.'s meta analysis of studies from affluent countries found 130 mins mean daily TV viewing, 34 mins mean computer use and 40 mins mean electronic game playing [28]. Our state-wide Queensland figures are similar, with daily mean daylight SBM times ranging from 83–123 mins depending on child age and sex [20]. Overall, media exposure of children (including reading comics and books, listening to radios and watching TV) appears to have been fairly stable over several decades [13]. However the use of computers and electronic games has doubled recently [29]. Children clearly have significant, and increasing, exposure to computers and electronic games.

Accompanying the increase in computer and electronic game use has been a concern about possible detrimental effects on children's health and development [29]. In a recent review [30], we reported that the available evidence suggested computer use targeted on learning areas is associated with enhanced academic achievement, but that electronic game playing has a negative effect on school achievement. We also found that game-related discourse may provide a stimulus for children's social development, although there are concerns about the potential negative effects of violence in electronic games.

Research on the impact of computer use on children's physical development has focused on postures during computer use at school, use of laptop computers and the impact of workstation design on posture and muscle activity [31]. This research has suggested potential musculoskeletal problems associated with prolonged and constrained postures and repetitive small movements. There is also some evidence that use of computers and electronic games can improve fine motor skills but decrease gross motor skills. The negative impact on gross motor development may be due to displacement of other childhood leisure activities which provide energy expenditure and critical practice of gross motor tasks [30].

The Australian guidelines for PA for children and adolescents [19], as well as prescribing 60 minutes of moderate to vigorous activity per day, recommend that not more than 2 hours per day, in daylight hours, is spent on SBM for entertainment purposes. This is defined as TV viewing, computer and video games and using a computer for non home-work purposes. The discussion paper [25] commissioned by the Australian government to facilitate the development of these guidelines recommended that 'children should avoid extended periods of inactivity through participation in sedentary activities such as TV, computer and video game usage and 'surfing the internet'. This aligns with the national guidelines from Canada to reduce 'non-active' time spent on electronic media by 30 minutes a day. The only other published recommendation referring to electronic media are those from the USA, which advocate that children watch less than 2 hours of TV per day [14,16], and recommend that children should avoid extended periods of inactivity. Our recent state-wide survey found 24% of Queensland children exceeded the national SBM guidelines, with the highest proportion (40%) reported by high school males [20]. Similarly, Hesketh et al. reported that by late elementary school, more than 80% of Victorian children did not meet the national guideline [32].

Whilst it is clear that children's use of SBM is substantial and growing, and that their use is often greater than national guidelines, the relationship between SBM use and PA is not clear. It has been suggested that TV viewing, computer use and electronic game playing may well have different relationships with PA or obesity and should therefore be investigated independently [33-35].

The majority of research investigating links between SBM exposure and PA has focused on TV viewing. There is extensive evidence linking TV viewing to increased body fatness (e.g. [36]), however, little that shows an effect on PA, except where TV viewing is contingent on activity. Whilst logic might suggest that TV viewing is related to less PA, the evidence to date for this relationship is surprisingly weak. The early evidence has not been consistently replicated. For example, Vandewater et al. and Burke et al. found no relationship between TV viewing and PA [37,34].

In a review of 39 studies in children and youth, Marshall et al. concluded that the relationship between TV viewing and PA is small (r = -.129) and negative [23], and this has been supported by similar reviews. Motl et al. recently reported a stronger relationship between naturally occurring fluctuations in TV viewing and PA [24]. However, only one RCT [38] has evaluated the effect of an intervention to reduce TV viewing on increasing PA. Whilst the study was able to demonstrate a reduction in body fatness, there was no effect on overall PA and it has not been replicated.

Whilst increased computer use has been associated with obesity in older girls [34], few studies have examined the link between computer use and PA. Vandewater et al. found a weak negative relationship between computer use and moderate PA [37] but Burke et al. found no relationship [33].

We have found a negative relationship between computer use and vigorous PA on weekends in 1600 young children [27]. This supported the work reported by Salmon et al. who found a negative correlation in 900 primary school children, between PA and having electronic games in the home, although time spent on electronic games was not measured [39]. Other studies have shown no associations between electronic game use and PA (e.g. [40,33]). More often than not electronic games are not singled out and are instead recorded together with general computer use and/or TV viewing, thus possibly distorting any true relationship. Marshall et al's review found an overall weak (r = -.141) negative correlation between electronic games or computer use and PA [23].

Currently there is no experimental evidence of a cause-effect relationship between electronic game use and overall PA. However, we have recently completed a pilot study with 12 children where their PA was compared between 8 week periods of having access to traditional electronic games and having no access to electronic games. Children wore Actical (Mini Mitter; Bend, OR) accelerometers for the final week in each condition. We found accelerometer assessed energy expenditure was reduced by 15% when children had access to traditional electronic games (no electronic games 1.07 kcal.min-1, traditional electronic games 0.91 kcal.min-1). Whist the pilot study lacked the power to detect the clinically significant effects, the trends support the hypothesis that playing traditional electronic games reduces overall PA and energy expenditure. This project will evaluate the effect of traditional electronic games on PA in a home-based RCT.

Current evidence for effect of electronic game exposure on PA may be limited because i) all electronic games have been grouped together or ii) the effect may differ between children.

In addressing the first issue, recently several studies have shown that PA during some electronic game playing can be significant with acceleration counts, energy expenditures and heart rates equivalent to activities such as skipping, jogging and stair climbing [41-44]. These studies used new domestic 'active' electronic game technology including dance mat, web camera and wireless controllers. [Dance mats are 1m2 with 9 pressure sensitive areas which respond to stepping – for images see . The games involve stepping on the correct square in time with music. EyeToy is a webcam which senses the arm, leg and trunk position of the user and shows the user's image 'within' the game – for images see . The games require the user to touch or avoid virtual objects. Wii uses 3D position and acceleration sensitive remotes to control bats and other game objects – see .].

**Figure 1:**
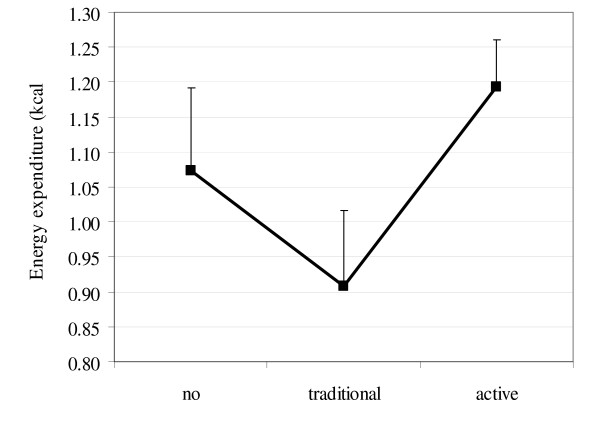
**Mean (SE) accelerometer assessed weekly energy expenditure during three electronic game access conditions**.

Therefore, just as there may be different effects of TV viewing, computer use and electronic game playing, the current weak evidence regarding the effect of electronic game playing on PA may be due to different effects depending on which type of electronic game is being played. However, the current evidence that some electronic games can be active is all laboratory based, and children may not use the active games sufficiently to impact on PA in the real world. In our recent pilot study we also compared the PA of children having 8 weeks access to active electronic games with 8 weeks access to traditional electronic games. We found accelerometer assessed energy expenditure to be 32% greater on non-school days when children had access to active electronic games (traditional e games 0.91 kcal.min-1, active e games 1.20 kcal.min-1; see Figure 1). Whilst the effect on school days was smaller (5%) the direction was consistent suggesting traditional and active game exposure may result in different overall PA outcomes. This project will evaluate the effect of active electronic games on PA in a home-based RCT.

The second reason why current evidence may be limited is that epidemiological studies have often used only age, sex and socioeconomic status as covariates in their models of the relationship between electronic game playing and PA when other variables may be equally or more influential.

Psychosocial variables are known to influence PA levels in children (e.g. perceived athletic competence [45]) and our research has found an impact on use of technology (e.g. children's experience of flow). The most widely used model of technology use (Davis' Technology Acceptance Model [46]) predicts that positive perceptions of technology increases the use of technology and this has been shown to be the case with computer use but has not been investigated with electronic game use. Biological variables are also known to influence PA by children. Children with poorer motor competence engage less in PA than other children [47] and overweight children spend more time with SBM [32]. Our pilot study data suggests the impact of electronic game access varied considerably between children. Therefore a further reason why current evidence is weak may be that sufficient appropriate covariates have not been added to epidemiological models. This project will explore a range of psychosocial and biological variables to inform future epidemiological studies.

The proposed mechanism for electronic game exposure to reduce PA is that it displaces more vigorous PA [25]. However there is to date little objective evidence for this, as electronic game use per se has rarely been specifically measured. Cummings et al., in a cross sectional study of 1490 adolescents, found that time spent on video-games was not correlated with time in active leisure for either boys or girls, but did correlate with less reading time [40]. Kautiainen et al. demonstrated that whilst TV viewing and computer use were positively correlated with overweight and obesity in adolescents, time spent playing digital games (video, computer and console games) was not associated with overweight and obesity [34]. Kautiainen et al. suggested that playing digital games may well be less sedentary than has been implied or is indeed simply related to a different lifestyle than viewing TV or using the computer [34]. This is supported by Olds et al. who have documented that certain children can combine high levels of sports participation with high SBM exposure, terming such children "technoactives" [48]. Similarly, Mutanga et al. found some children with high SBM use also had more PA [49].

Rey-Lopez et al. in their review of sedentary behaviour and obesity, conclude that there is a need for methodologically stronger studies to investigate whether (and what type of) sedentary behaviour displaces PA, and how sedentary behaviour influences health outcomes [35]. Our pilot study results suggest that electronic game use displaces some sedentary activities (watching TV, reading books) and some active leisure. We also propose that displacement patterns will be different depending on the nature of the technology (sedentary/active). Whether or not electronic games have an impact on overall PA, their impact on other activities needs to be understood. This project will examine the activities displaced by children when they have access to electronic games.

In summary, there is clearly a need to provide better evidence on this priority health issue [40,25,50]. Specifically whether there is a cause-effect relationship between electronic games and PA needs to be tested experimentally, and the role of potential confounds related to the type of electronic game played and characteristics of the child need to be considered. Finally, the displacement of other leisure activities by electronic game playing needs to be understood.

## Methods/Design

### Design and Aims

This study will use a randomised and controlled trial to assess the impact of electronic game use on PA in children by:

1) comparing PA and energy expenditure where children have either no electronic games or access to traditional electronic games. We hypothesise that PA and energy expenditure will be reduced when children have access to traditional electronic games.

2) comparing PA and energy expenditure where children have access to either traditional electronic games, new active electronic games and no electronic games. We hypothesise that PA and energy expenditure will be greater with new active electronic games, but still less than no games.

3) examining responses of different children to explore whether the impact of access to electronic games is greater in some children. We hypothesise a stronger effect on children with poor coordination skills, high adiposity, poor social confidence, more positive attitudes to technology and less positive attitudes to PA.

4) examining displacement of sedentary and active leisure activities by electronic games. We hypothesise that all electronic games will displace active non-electronic leisure activities.

### Participants

36 boys and 36 girls (10–12 years of age) will be progressively recruited by mass media, community newsletters and local school notices. This age group has been selected as they are able to provide detailed information in diary and questionnaires, have a high use of electronic games and are developing activity patterns pre-puberty which may track into adulthood. Recruitment will be staggered over three years and targeted to enable participation of equal numbers of males and females, and children representative of a spread of socio-economic status, electronic game experience and motor competence Volunteers will be screened to ensure they are willing to participate after being informed of the full study responsibilities and meet the inclusion and exclusion criteria. Inclusion criteria are: aged 10–12 years at start of study and able to use electronic games on most days. Children will be excluded if they have a diagnosed disorder likely to impact their study participation, movement or electronic game use (other than developmental coordination disorder), live in a shared care arrangement where the child spends a significant amount of time in different houses and is unable to maintain game access condition, or live remote to the University campus. In 2009, 12 boys and 12 girls will be tested, with equal numbers tested 2010, and 2011.

For power calculations, daily moderate/vigorous PAL was estimated at 115+30 mins with a minimum effect size of 15 mins considered important based on effects in prior studies by us and others [51]. If the variation in the PA level between repeated time points in each individual is normally distributed with standard deviation 30 mins, and the true effect of game condition is 15 mins, a study with 72 subjects will reject the null hypothesis that this response difference is zero with probability (power) 0.986. The Type I error probability associated with this test of this null hypothesis is 0.05. If the Type I error is lowered to 0.01 to account for 'repeated' contrasts between conditions, the power is 0.943 [52]. We [51] have had a compliance rate of 95% for DLW data collection and 92% for accelerometry, and both measures have been widely used in studies of 50 to 100 children [53,9]. We have allowed for 10% attrition in data.

Volunteers and their parents were provided a detailed written description of the study purpose, procedures, risks and benefits and given an opportunity to ask research staff for clarification prior to signing assent (children) and consent (parents) to participate. The study has ethical approval from the Human Research Ethics Committee of Curtin University of Technology (approval number HR131/2006).

### Intervention and control conditions

There will be three levels of electronic game access. 'No electronic games' will involve all electronic games removed from the family home with a contract that electronic games will be avoided where possible at other locations. 'Traditional electronic games' will involve the provision of a Sony PlayStation 2 with a range of non-violent games requiring game pad input. 'Active electronic games' will involve the provision of a Sony PlayStation 2 with EyeToy and dance mat input devices and a range of non-violent games. A condition period of 8 weeks has been found sufficient to show physical and psychological changes. It also allows for children to accommodate to each condition and is not so long to adversely affect compliance in the 'no games' condition. From our pilot study and discussions with 10 year olds, the removal of all electronic games will be acceptable as a way of getting access to a range of new games and equipment for four months. This is why a within subjects design is required.

### Outcome measures

#### Physical activity

Time spent in moderate to vigorous intensity PA, as well as total movement, will be assessed over 7 days using two Actical accelerometers worn on the wrist and the hip. The MiniMitter Actical is the most widely used and validated accelerometer in studies of children and adolescents [54,9]. Seven days of accelerometer measurement are recommended for the purposes of acceptable measurement of moderate to vigorous PA [9]. Total weekly PA as well as weekend PA and after school weekday PA will be assessed.

#### Energy expenditure

Total energy expenditure (TEE) will be measured using the DLW technique [55,56]. This is the gold standard method for assessing free living TEE and has been used extensively in children and adolescents [57]. Due to the nature of the study with children being assessed in the home at different points in the day, in non-fasted states, a measured RMR estimate is not deemed a suitable. BMR will therefore be predicted from the subjects sex, age, weight and height using Schofields's equations for children aged 10–17 yrs of age [58]. Predicted BMR has been shown to have good agreement with measured RMR by indirect calorimetry in children of all ages [59,60].

From these two energy expenditure measurements, the habitual physical activity level (PAL) is calculated as the ratio of TEE: BMR. The PAL ratio is a convenient way of adjusting energy expenditure for age, sex, weight and body composition and is a widely accepted measure of habitual PA [61,62]. The DLW technique involves collection of a daily urine sample for 10 days. These samples will be stored in Perth, and sent to Brisbane for subsequent analysis.

#### Motor coordination

Motor competence will be assessed using Movement Assessment Battery for Children-2 (MABC-2) [63]. The MABC-2 comprises 8 tasks, three measuring manual dexterity, 3 measuring aiming and catching and 2 measuring balance. Age norms based on a standardisation sample of 1,172 children are used to determine a standard total score (*M *= 10, *SD *= 3). Separate standard scores can be determined for each of the sub-tests. In addition to the total score, a set of qualitative observations allows the examiner to record the child's performance characteristics during the testing. Cut-offs for impairment scores are at or below the 5th percentile for definite motor difficulties, whilst scores above the 5th percentile but below the 15th percentile suggest borderline difficulties. Minimum value of the test-retest reliability of the original MABC is 0.75 and the inter-tester reliability is 0.70. The original MABC has been found to correlate well with other movement tests [64,65]. Assessment of MABC2 will be conducted by a second research officer blind to participant condition.

#### Adiposity

Percentage body fat will be determined from the measurement of the ^18^O dilution space, which is calculated as part of the DLW technique for measuring energy expenditure. Taking into account the fact that ^18^O overestimates total body water by 1% [66], and using published total body hydration constants from children of different ages [67], fat-free mass can be calculated from total body water. Fat mass is calculated as the difference between total body weight and fat-free mass and expressed as a percentage.

Waist circumference measurements will be taken and age and sex adjusted BMI z-scores will be calculated to enable comparison with state-wide surveys (WA, QLD).

#### Child Self-Perceptions

Harter's Self-Perception Profile for Children [68] will assess self-perceptions across domains of Scholastic Competence, Social Acceptance, Athletic Competence, Physical Appearance and Behavioural Conduct, and it also includes a subscale designed to evaluate global self-worth that assesses self-esteem independent from the competence domains. It has been validated in samples of children from a wide range of cultural backgrounds, including in Australian children and has high levels of internal consistency ranging from .74 to .92 [68].

#### Attitudes to technology

Attitudes to technology will be assessed using Webster et al's measure of 'flow' which we have previously shown to have acceptable reliability when used with children aged 10–12 years (Cronbach's alpha .82) [69]. In addition we will use Deane et.al's measures to assess the Technology Acceptance Model factors of 'ease of use' and 'usefulness of technology' which show acceptable to good internal reliability (.67 and .82) [70].

#### Attitudes to PA

Attitudes to PA will be assessed using the revised Physical Activity Enjoyment Scale [71]. Internal consistency, test-retest reliability and construct validity has been demonstrated [72-74].

#### Leisure activity

To provide descriptive information on the type of activities performed, participants will use a modified version of the previous-day PA recall (PDPAR) in the form of a diary for 7 days. In the PDPAR the predominant activity for 30 minute blocks during waking hours is recorded. Use of the PDPAR over several consecutive days, in the form of a diary has also been shown to be valid, against measures of accelerometry, and feasible [75]. From this diary, displacement of activity categories will be calculated.

### Covariates

#### Season

Prior PA research has identified significant differences between summer and winter seasons and interactions with sex (more reduction in PA in winter in girls) [21]. The potential seasonal effect will be allowed for in the design by having a balanced ordering of game conditions and a staggered start to cover the school year.

#### Electronic game experience

Computer and electronic game and interface experience which could confound the effect of the game condition will be measured using a questionnaire based on our prior studies and a large USA study [29].

#### Sex

Equal numbers of boys and girls will participate. Boys and girls are known to differ in the nature of their electronic game exposure and in the other measures being taken. The games selected will be based on discussions with children to ensure desirability by both girls and boys, as per our successful pilot study.

### Procedure

Following screening, participants will be randomly allocated to an order of conditions by selection of an opaque sealed envelope. A balance of orders across the year will be achieved by having 4 sets of the 6 possible order permutations in each year cohort of 24, repeated over three years. After informed consent/assent from parent and child, a research officer (RO) will visit the home and instruct parent and child in baseline assessments. This visit will include measurement of resting energy expenditure, explanation of the DLW method and initial dosing, explanation of the assessment and questionnaire. The RO will return after 10 days to collect baseline assessments and set up the electronic game condition. This will involve either removal of all electronic games or setting up electronic game equipment and instructing parent and child in its use. Follow-up phone calls will be made the next day and after 6 days to ensure game equipment is working correctly. Towards the end of the 6th week in a condition the RO will visit again to set up the DLW and PA assessments. After 8 weeks the RO will collect DLW samples and completed measures and set up the next condition. After all 3 conditions a debriefing interview with parent and child will be conducted to gain qualitative details on the trial. The family structure including number, age and sex of siblings will be recorded, and the behaviour of siblings during the trial will be assessed at debriefing interview. Assessment will be scheduled to avoid school and public holidays where possible. Individualised reports will be provided to participants.

### Trial flow

**Figure 2:**
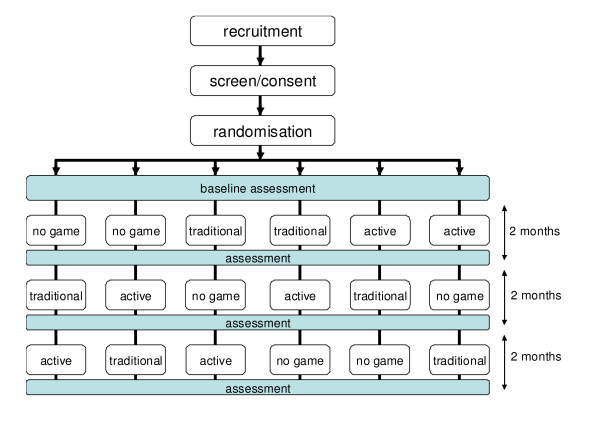
**Trial flowchart**. This figure provides an overview of the participant flow.

Figure 2 provides an overview of the trial flow. Following recruitment, screening and consent, participants are randomised to an order of electronic game conditions. After baseline assessments participants are setup in their first condition and are assessed at the end of the 8 week condition. Participants are then set up in their second condition for 8 weeks and then their third condition for 8 weeks, again with assessment occurring at the end of each condition.

### Analysis

To examine hypotheses 1 (that PA and energy expenditure will be reduced when children have access to traditional electronic games) and 2 (that PA and energy expenditure will be greater with new active electronic games, but still less than no games) a 4 (baseline and 3 game conditions) × 2 (sexes) repeated measures ANCOVA analysis with pre-specified contrasts will be conducted for each PA and energy expenditure variable. Prior game experience will be used as a covariate. A critical alpha level of 0.01 will be used to balance type 1 and type 2 errors.

To assess hypothesis 3 (that there will be a stronger effect on children with poor coordination skills, high adiposity, poor social confidence, more positive attitudes to technology and less positive attitudes to PA), separate multiple linear regression analyses for both traditional sedentary electronic and new active electronic games will examine the importance of individual characteristics on activity levels (total minutes of overall moderate/vigorous PA). Sex and amount of moderate/vigorous PA at the end of the no game period will be included as baseline covariates for the assessment of the independent effect on PA of; motor competence, adiposity, perceived athletic competence and attitudes to technology. To assess if individual characteristics may impact differently upon the relationship between PA and traditional sedentary electronic versus PA and new active electronic games, beta coefficients and associated 95% confidence intervals, and proportions of total variance explained by these variables, will be compared between the two models.

To assess hypothesis 4 (that all electronic games will displace active non-electronic leisure activities) the impact of the three study conditions on time spent in four outside school activities (reading, watching TV, using computers, playing sports and non-organised physical activity) will be examined using a 3 (no electronic games, traditional electronic games, active electronic games) ×2 (sex) repeated measures MANOVA with weekly minutes in each activity as the dependent variables.

## Discussion

Increasing PA is a major health priority internationally due to the associated health burden. It is widely believed that access to electronic games decreases PA in children. Whilst there is considerable research on the impact of TV viewing on PA and obesity, there is little research specific to electronic games. The available evidence suggests electronic games and TV have different associations with PA. Playing electronic games is an important discretionary leisure activity for many children, yet the impact of this on PA has not been tested experimentally. Further, the influence of different game technologies and child characteristics on the impact is not known. Finally, whilst it is assumed playing electronic games displaces more vigorous activities, this has not been demonstrated. As far as we know, this randomised and controlled trial will be the first to experimentally evaluate the impact of access to electronic games in the home environment on children's PA.

### Implications

This trial will provide critical information to understand whether access to electronic games effects children's PA. Given the vital importance of adequate PA to a healthy start to life and establishing patterns which may track into adulthood, this project can inform interventions which could have a profound impact on the long term health of children.

## Competing interests

The authors declare that they have no competing interests.

## Authors' contributions

All authors have contributed substantially to this protocol. LMS conceived the study, contributed to the study design and drafted the manuscript. RAA, JPP, CMP, PSD and AJS contributed to the study design and revised the manuscript. All authors have read and approved the final manuscript.

## Pre-publication history

The pre-publication history for this paper can be accessed here:


